# Quantitative neuronal morphometry by supervised and unsupervised learning

**DOI:** 10.1016/j.xpro.2021.100867

**Published:** 2021-09-30

**Authors:** Kayvan Bijari, Gema Valera, Hernán López-Schier, Giorgio A. Ascoli

**Affiliations:** 1 Center for Neural Informatics, Structures, & Plasticity and Neuroscience Program, Krasnow Institute for Advanced Study, George Mason University, Fairfax, VA 22030, USA; 2 Sensory Biology and Organogenesis, Helmholtz Zentrum Munich, 85764 Neuherberg, Germany; 3 Bioengineering Department, Volgenau School of Engineering, George Mason University, Fairfax, VA 22032, USA

**Keywords:** Bioinformatics, Cell Biology, Microscopy, Neuroscience, Computer sciences

## Abstract

We present a protocol to characterize the morphological properties of individual neurons reconstructed from microscopic imaging. We first describe a simple procedure to extract relevant morphological features from digital tracings of neural arbors. Then, we provide detailed steps on classification, clustering, and statistical analysis of the traced cells based on morphological features. We illustrate the pipeline design using specific examples from zebrafish anatomy. Our approach can be readily applied and generalized to the characterization of axonal, dendritic, or glial geometry.

For complete context and scientific motivation for the studies and datasets used here, refer to Valera et al. (2021).

## Highlights

• Quantification of microscopic neural tracings stored in SWC format
• Unsupervised and supervised analysis of quantified features of neural morphologies
• Identification of morphological signatures differentiating groups of neural arbors
• Open-source software that can be modified, expanded, and applied to diverse problems

## Graphical abstract

**Figure:**
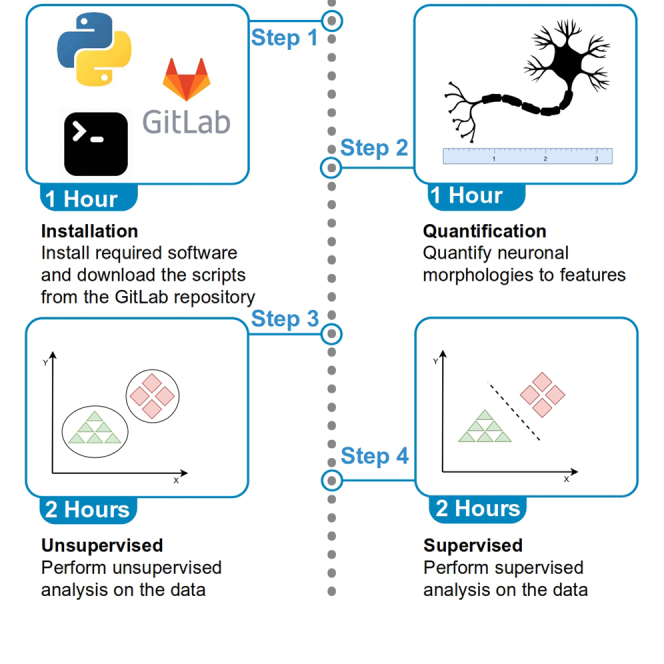

## Before you begin

Technical details of data acquisition, imaging modalities and neuronal tracing are discussed in depth in the original publication (Valera et al., 2021). Continuous advances in both microscopy and computational power are making the semi-automated reconstructions of neuronal arbors progressively more practical (Peng et al., 2017). This protocol describes how to quantify digitized neurons into descriptive morphological features and then apply unsupervised, supervised, and statistical analysis on quantifiable morphological attributes, see also (Polavaram et al., 2014). The software setup described here was deployed on Ubuntu 20.04 LTS Linux distribution. However, all referred tools and packages function properly across different platforms, including Windows 10.

### Installation of analysis tools and downloading datasets and custom codes

**Timing: 1 h**

1. Install L-Measure v5.3 or v5.2 software (depending on the available version for your operating system).
  a. Download the version relevant to your platform. More information in this regard is available on the L-Measure website (see key resources table for the research resource identifier (Bandrowski et al., 2016)).
2. Install Python 3.8 on your computer system (see key resources table for link) or on a virtual environment (see Note below).
  a. Install the following packages on your installed python:
    i. matplotlib version 3.3.4
    ii. NumPy version 1.20.1
    iii. Pandas version 1.2.2
    iv. scikit-learn version 0.24.1
    v. SciPy version 1.6.0
  b. Alternative 1: After activating your virtual environment from the ‘scripts’ directory, use ‘pip install -r requirements.txt’ to automatically install all required packages.
  c. Alternative 2: We have provided ‘.yaml’ file located in the ‘scripts’ directory to install both python environment and the required packages. To do so, open your ‘conda’ terminal and run ‘conda env create --name star-protocol -f star-protocol.yaml’. This will create the python environment named ‘star-protocol’ along with the right packages. For more information about ‘conda’ and its installation, please visit: https://conda.io/projects/conda/en/latest/user-guide/index.html

***Note:*** After installing Python, all scripts can be run either via command line using ‘**python [name-of-script].py [input parameters]**’ or through any preferred python environment interface providing optimized utilities for code modifications.

3. Download from the GitLab repository all python scripts and representative data pertaining to this protocol and associated use case scenario.
  a. URL: https://gitlab.orc.gmu.edu/kbijari/zebrafish-analysis-protocol
4. For simplicity purposes, all reconstruction files used in the examples illustrated here are also available in this GitLab repository. The reconstruction files from this and thousands of additional datasets can also be obtained from NeuroMorpho.Org (Ascoli et al., 2007).

## Key resources table

**Table:** 

REAGENT or RESOURCE	SOURCE	IDENTIFIER
**Deposited data**		

Neuronal reconstructions	NeuroMorpho.Org	RRID:SCR_002145
Source code	gitlab.orc.gmu.edu/kbijari/zebrafish-analysis-protocol	RRID:SCR_021638

**Software and algorithms**		

Python 3.8	python.org/downloads/	RRID:SCR_008394
L-Measure	cng.gmu.edu:8080/Lm	RRID:SCR_003487
SciPy 1.6.0	scipy.org	RRID:SCR_008058
scikit-learn 0.24.1	scikit-learn.org	RRID:SCR_002577
Pandas 1.2.2	pandas.pydata.org	RRID:SCR_018214
NumPy 1.20.1	numpy.org	RRID:SCR_008633
matplotlib 3.3.4	matplotlib.org	RRID:SCR_008624

## Step-by-step method details

We begin by illustrating how to extract quantitative morphological attributes from digital reconstructions of neurons represented in the standard SWC file format (Nanda et al., 2018). Then, we demonstrate the procedure to run unsupervised, supervised, and statistical analysis on these attributes.

### Data quantification

**Timing: 2 h**

**Figure 1:**
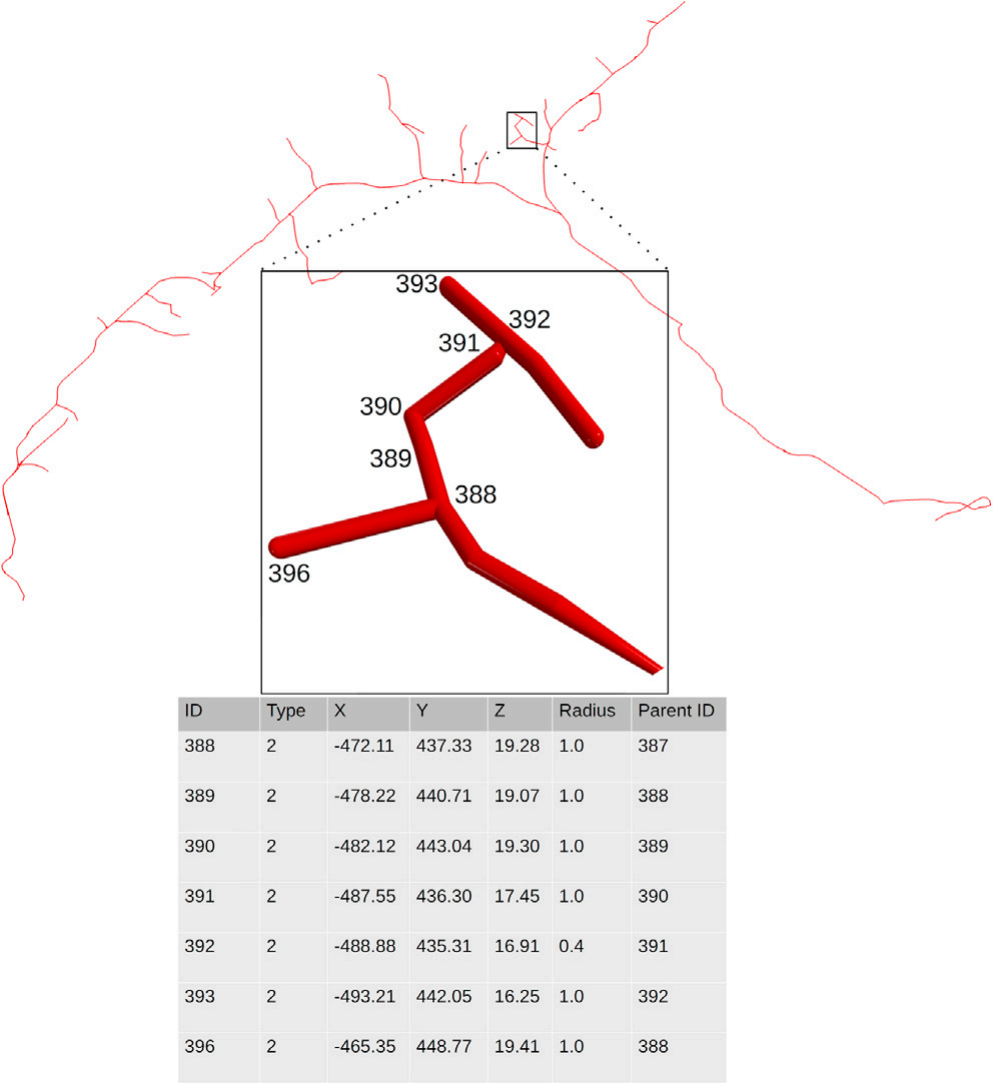
Sample visualized neuron from the zebrafish data along with the corresponding rows of its SWC file

These steps describe how to process and quantify neural morphologies stored in the SWC file format (Figure 1). These are most typically representing neurons and glia (Abdellah et al., 2018; Ascoli et al., 2017) but have also been used for vascular reconstructions (Wright et al., 2013). The output is a set of quantitative morphological attributes for every neuron, which could be used for supervised, unsupervised, or any other quantitative analysis.

**Figure 2:**
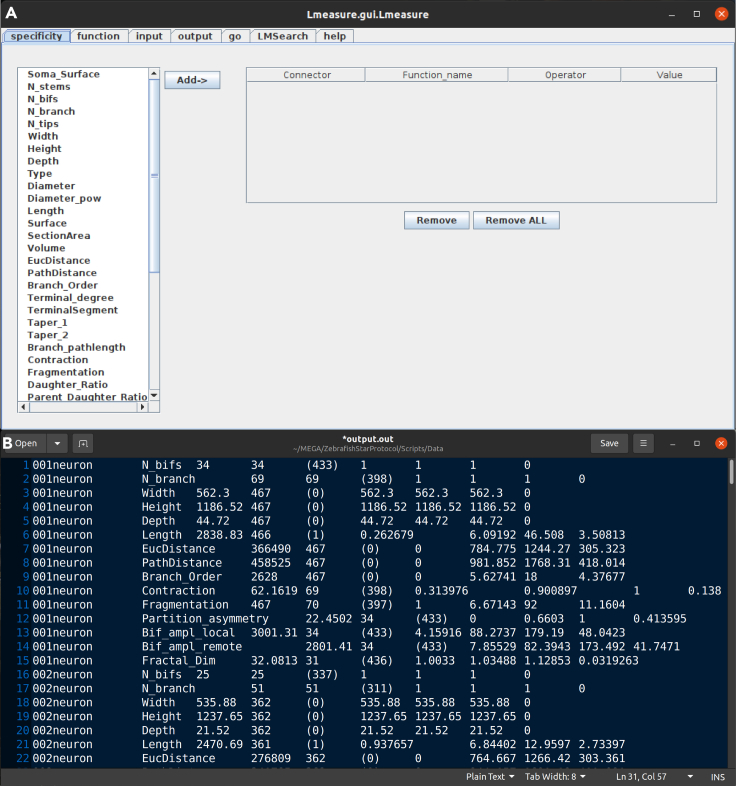
Neuronal quantification (A) Graphical User Interface of the L-Measure software (open on the default ‘specificity’ tab). (B) Sample output file produced by L-Measure.
2. In the ‘*function*’ tab select the morphological attributes that you wish to extract from the SWC files. The selected morphological attributes utilized here (referred to henceforth as “core metric functions”) are: 'N_bifs', 'N_branch', 'Width', 'Height', 'Depth', 'Length', 'EucDistance', 'PathDistance', 'Branch_Order', 'Contraction', 'Fragmentation', 'Partition_asymmetry', 'Bif_ampl_local', 'Bif_ampl_remote', 'Fractal_Dim' (see also Note below).

***Note:*** The ‘included’ and ‘excluded’ points in step 6 are not user-defined, but rather automatically determined by L-Measure and displayed for information only. Specifically, these values refer to the number of rows in the SWC file used to calculate the desired morphometric. For instance, the functions measuring the bifurcation angles (Bif_ampl_local and Bif_ampl_remote) only operate on the arbor bifurcation points, and not on the stems, continuations, and terminations. Thus, the included points would be in this example the counts of bifurcations and the excluded points would be the rest of the arbor tracing points. For exact definitions and more information about core metric functions and the measurements extracted in Step 2, visit the L-Measure website (http://cng.gmu.edu:8080/Lm/help/index.htm).

1. Run L-Measure in your operating system.
  a. In Linux you need to run **java -jar Lm.jar** in the command line from the folder where the L-Measure executable scripts are located.
  b. Check the troubleshooting section of the L-Measure website if you run into errors.
  c. Figure 2 shows the main view of L-Measure software being executed and related sample output.

3. In the ‘*input*’ tab select the SWC files corresponding to one or more neurons from the local drive.
4. In the ‘*output*’ tab specify a file name and the location where you wish to store the extracted values.
5. Finally, in the ‘*go*’ tab press ‘go’ for L-Measure to run the analysis and calculate the numerical attributes for each SWC file.
6. In the output file, L-Measure produces neuron name, name of the core metric function, total sum of the function over all tracing points (Total_Sum), number of points included in the analysis (Count_considered_compartments), number of points excluded (Count_discarded_compartments), minimum value (Min), average (Ave), maximum (Max), and standard deviation (S.D.) of each metric function (Scorcioni et al., 2008).
7. Edit these quantifications by removing irrelevant or redundant statistics in each row and save the resultant file in comma separated value (CSV) format (see Note below for more details). Every line in this CSV file should represent a single neuron. File ‘neuron_features.csv’ in the scripts/data folder was obtained in that way from the output of L-Measure for this experimental dataset. Alternatively, you can run ‘python convert.py -i /location/of/input-file -o /location/to/save/output-file.csv’; this script reads the results yielded fromL-Measure from the user’s specified location and creates a CSV file (output-file.csv) that can be used for both the supervised and unsupervised subsequent steps.

***Note:*** Step 7 is required because not all elements of the L-Measure default statistical summary are appropriate for every feature. Therefore, selecting the most suitable statistic for the analysis is important. For more information on the used parameters and their relevance see Table 1 below. It is important to mention that L-Measure does not calculate median values and mean-based statistics may not be appropriate if the data is not normally distributed. A detailed description of the values L-Measure can extract from the SWC files and their applications is provided by the reference paper (Scorcioni et al., 2008).

**Table 1:** Description of L-Measure outputs

Core function (brief description)	Relevant statistics to consider	Reasoning behind the chosen statistic
N_bifs (number of bifurcations)	Total_sum	Returns the total number of bifurcations
N_branch (number of branches)	Total_sum	Returns the total number of branches
Width (neuronal width)	L-Measure returns the same value for Min, Max, and Ave	Horizontal extent (x-coordinate) containing 95% of all tracing points
Height (neuronal height)	L-Measure returns the same value for Min, Max, and Ave	Vertical extent (y-coordinate) containing 95% of all tracing points
Depth (neuronal depth)	L-Measure returns the same value for Min, Max, and Ave	Depth (z-coordinate) containing 95% of all tracing points
Length (total arborization length)	Total_sum	Returns the total length summed across all compartments
EucDistance (maximum Euclidean distance from soma to the tips)	Ave and max are relevant, we used Max	Maximum straight distance encompassing the whole neuron
PathDistance (path distance of a compartment)	Ave and max are relevant, we used Max	Maximum geodesic distance from soma to tips
Branch_Order (order of the branch with respect to the soma)	Ave and max are relevant, we used Max	Maximum number of bifurcations from soma to tips
Contraction (ratio between Euclidean distance of a branch and its path length)	Ave	Average tortuosity across all branches
Fragmentation (total number of compartments that constitute a branch between two bifurcation points)	All are relevant, we used Total_sum	Total number of compartments from all of the branches
Partition_asymmetry (average over all bifurcations of sub-trees)	Ave	Topological tree asymmetry measured from all bifurcation points
Bif_ampl_local (angle between the first two bifurcation compartments)	All are relevant, we used Ave	Average over all bifurcations of the angle between the first two daughter compartments
Bif_ampl_remote (angle between, current plane of bifurcation and previous plane of bifurcation)	All are relevant, we used Ave	Average over all bifurcations of the angle between the following bifurcations or tips
Fractal_Dim (slope of linear fit of regression line obtained from the plot of path vs. Euclidean distances)	All are relevant, we used Ave	Average space occupancy measured from all branches

### Unsupervised clustering

**Timing: 2 h**

The following steps describe the unsupervised analysis, a process that groups neurons based on their morphological features independent of any *a priori* knowledge about the cells. We start with the fundamental K-means algorithm for data clustering and then use graphical mixture models to find innate distributions within the dataset.

8. From your Python environment or command line, run ‘python unsupervised-elbow-curve.py -i ./Data/neuron_features.csv’.
  a. This script reads the neuronal data from the data folder and normalizes them.
  b. Then it plots the elbow curve, which shows the trade-off between residual variance and the number of clusters (Figure 3A).

**Figure 3:**
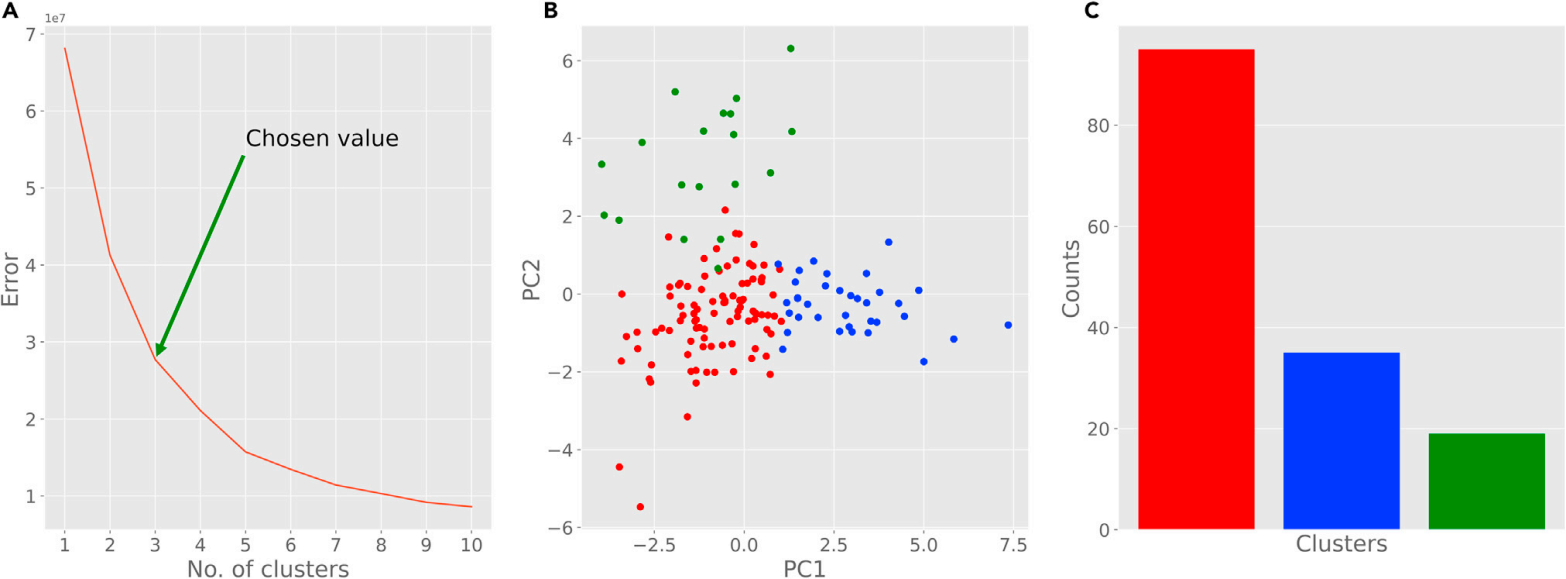
K-means clustering results (A) Elbow curve to determine the optimal number of clusters. (B) Scatter plot of the neurons based on their first two principal components (PC1 and PC2) and color-coded clusters (each color represents a cluster). (C) Distribution of different cluster assignments found by K-means algorithm.
  c. Elbow curve is used to determine the optimal number of clusters. This heuristic method calculates the sum of squared distances of all points from the center of their respective cluster as a function of the number of clusters. The point(s) of maximum inflection (visible bends) are appropriate choices for the number of clusters. In this case, the curve has two high-inflection points (3 and 5) and we selected 3 as the number of clusters for our analysis.
9. From your Python environment or command line, run ‘python unsupervised-kmeans.py -i ./Data/neuron_features -k 3’.
  a. This script reads the neuronal data from the data folder and groups them into 3 clusters utilizing the K-means algorithm.
  b. The script prints the cluster label for each neuron as well as the centers of the three clusters.
  c. The program also performs a principal component analysis to optimize the spatial display of the feature distribution along the main directions of their variance.
  d. The program then plots (i) a scattered visualization of the data based on the first two principal components and (ii) a bar plot depicting the count of neurons in each cluster (Figures 3B and 3C).
  e. You can alter the number of clusters by changing the input parameter “k” before running the script.
10. From your Python environment or command line, run ‘**python unsupervised-BIC-curve.py -i ./Data/neuron_features.csv**’.
  a. This script reads the neuronal data from the data folder and normalizes them.
  b. Then it plots the Bayesian Information Criterion (BIC) and Akaike Information Criterion (AIC) curves, which quantify the grouping distinctiveness as a function of the number of clusters (Figure 4A).

**Figure 4:**
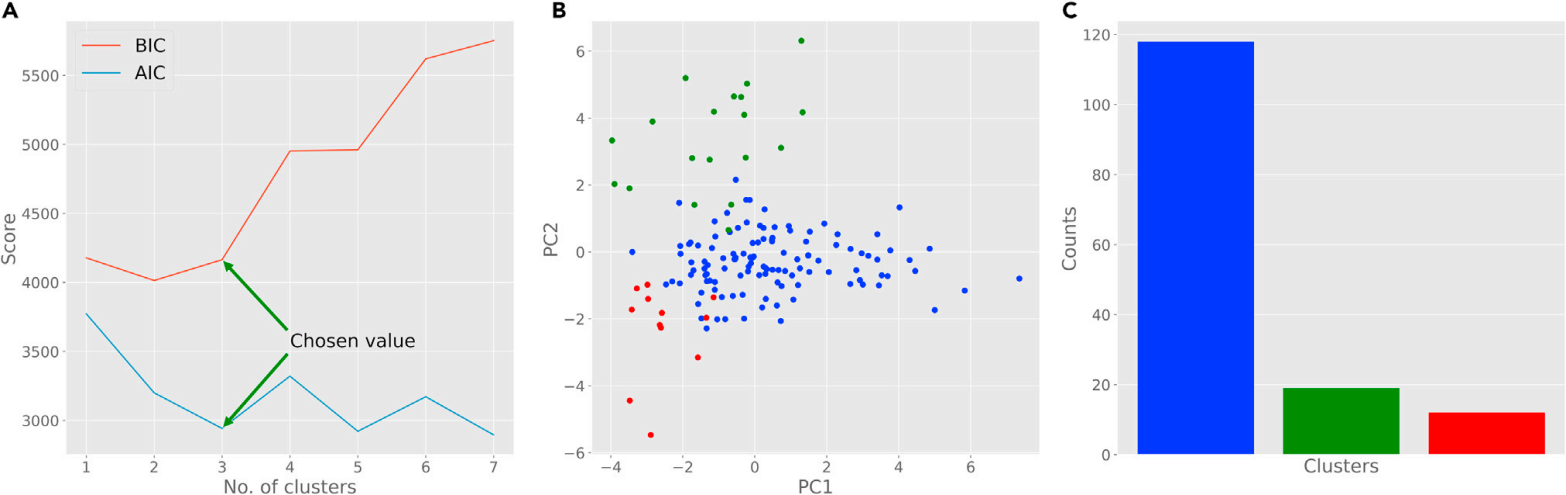
Gaussian Mixture Model (GMM) clustering results (A) Bayesian Information Criterion (BIC) and Akaike Information Criterion (AIC) scores to determine the optimal number of clusters. (B) Scatter plot of the neurons based on their first two principal components (PC1 and PC2) and color-coded clusters (each color represents a cluster). (C) Distribution of different cluster assignments found by GMM.
  c. Bayesian Information Criterion (BIC) is a likelihood-based method for model selection among a finite set of possibilities. This is a heuristic score to determine the optimal number of clusters.
  d. Akaike Information Criterion (AIC) measures the relative amount of information lost by a given model for a certain number of clusters.
  e. Generally, lower values of AIC and BIC are more desirable. For our analysis, we selected 3, where both curves have relatively low value compared to neighboring points.
11. From your Python environment or command line, run ‘python unsupervised-GMM.py -i ./Data/neuron_features.csv -k 3’.
  a. This script reads the neuronal data from the data folder and groups them into 3 clusters using Gaussian mixture distributions.
  b. The script prints the group label for each neuron.
  c. The program also performs a principal component analysis to optimize the spatial display of the feature distribution along the main directions of their variance.
  d. The program then plots (i) a scattered visualization of the data based on the first two principal components and (ii) a bar plot depicting the label distribution (Figures 4B and 4C).
  e. You can alter the number of clusters by changing the input parameter “k” before running the script.

***Optional:*** Scikit-learn includes various algorithms to perform grouping and clustering datasets. Based on the nature and inherent characteristics of the data, different clustering models might achieve more interpretable outcomes (Bijari et al., 2018). To explore different clustering techniques please visit https://scikit-learn.org/stable/modules/clustering.html for an overview of suitable alternatives and code snippets to execute them.

### Supervised clustering

**Timing: 2 h**

Many datasets of interest, including the examples employed here, have specific labels assigned to each neuron, representing knowledge that the researcher has about these cells that is in principle unrelated to the arbor morphology. Examples of such labels (often referred to as “true classes”) include electrophysiological characteristics, anatomical location of the soma, expression of particular genes, functional specificity, and many more (Ascoli and Wheeler, 2016). Having the data labels in hand, the next series of steps describes the process of supervised analysis. This process assesses the morphological features that best separate among the chosen labels. We begin with visualizing the dataset to gain a spatial perception of the different data points based on their principal components. Then we add the labels to the results of the previous *unsupervised* methods to check whether the intrinsic morphological characteristics of the cells, as discovered by unsupervised learning, correspond to any independent functional property (distinct ground truth classes) and ascertain how adequately the supervised algorithms perform relative to the known information. At the end, we train and test a battery of *supervised* classification methods on the labeled data to quantify how accurately the morphological features can discern among the labels.

12. For this step, neural data should have explicit class labels. There are typically no limits to the number of these labels (ground truth information) and they are typically determined by the experimentalist while preparing the dataset based on biologically relevant knowledge. To prepare your labels, please create or format your data labels as described below.
  a. Use ‘labels (template).xlsx’ in the ‘Scripts’ folder and label each neuron with the proper label based on your experiment.
  b. If you have more than one class, use additional columns.
  c. Save the template file for your records. Also, export a CSV file to provide input for the next steps in the analysis.
13. From your Python environment or command line, run ‘python supervised-visualize.py -i ./Data/neuron_features.csv -l ./Data/neuron_labels.csv’.
  a. This script reads the neuronal data and their corresponding labels from the data folder.
  b. The program next performs a principal component analysis to optimize the spatial display of the feature distribution along the main directions of their variance.
  c. The program then outputs a series of scatter plots, one for each set of labels, with each neuron data point associated with its label symbol (Figure 5).

**Figure 5:**
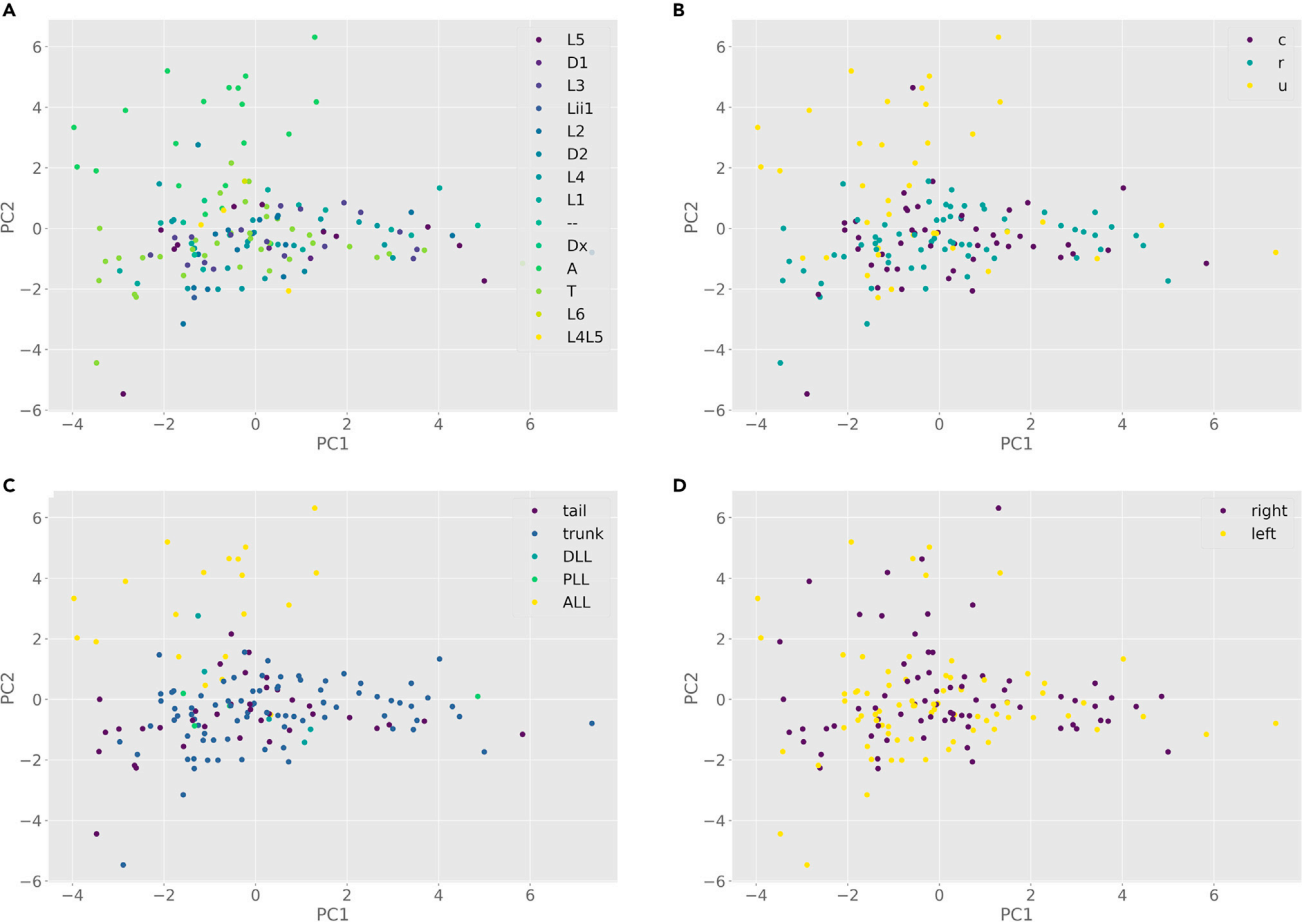
Distribution of the neurons based on their principal components (PC1 and PC2) and their ground truth labels (A) Scatter plot based on different neuromast labels (A: anterior, L: lateral, T: trunk, D: dorsal; numbers associated with the labels indicate closeness of the neuron to the head of the animal, with 1 being the closest and 6 being furthest). (B) Scatter plot based on different tuning labels (u: unknown, r: rostral, c: caudal). (C) Scatter plot based on different region labels (trunk, tail, posterior lateral line, dorsal lateral line, and anterior lateral line). (D) Scatter plot based on different hemisphere labels (right and left).
14. From your Python environment or command line, run ‘python labeled-kmeans.py -i ./Data/neuron_features.csv -l ./Data/neuron_labels.csv -k 3’.
  a. This script reads the neuronal data and groups them into 3 clusters.
  b. The program then outputs the homogeneity score and a series of scatter plots, each displaying the color-coded K-means clusters with symbols (shapes) corresponding to the true class labels in each label category (Figure 6).

**Figure 6:**
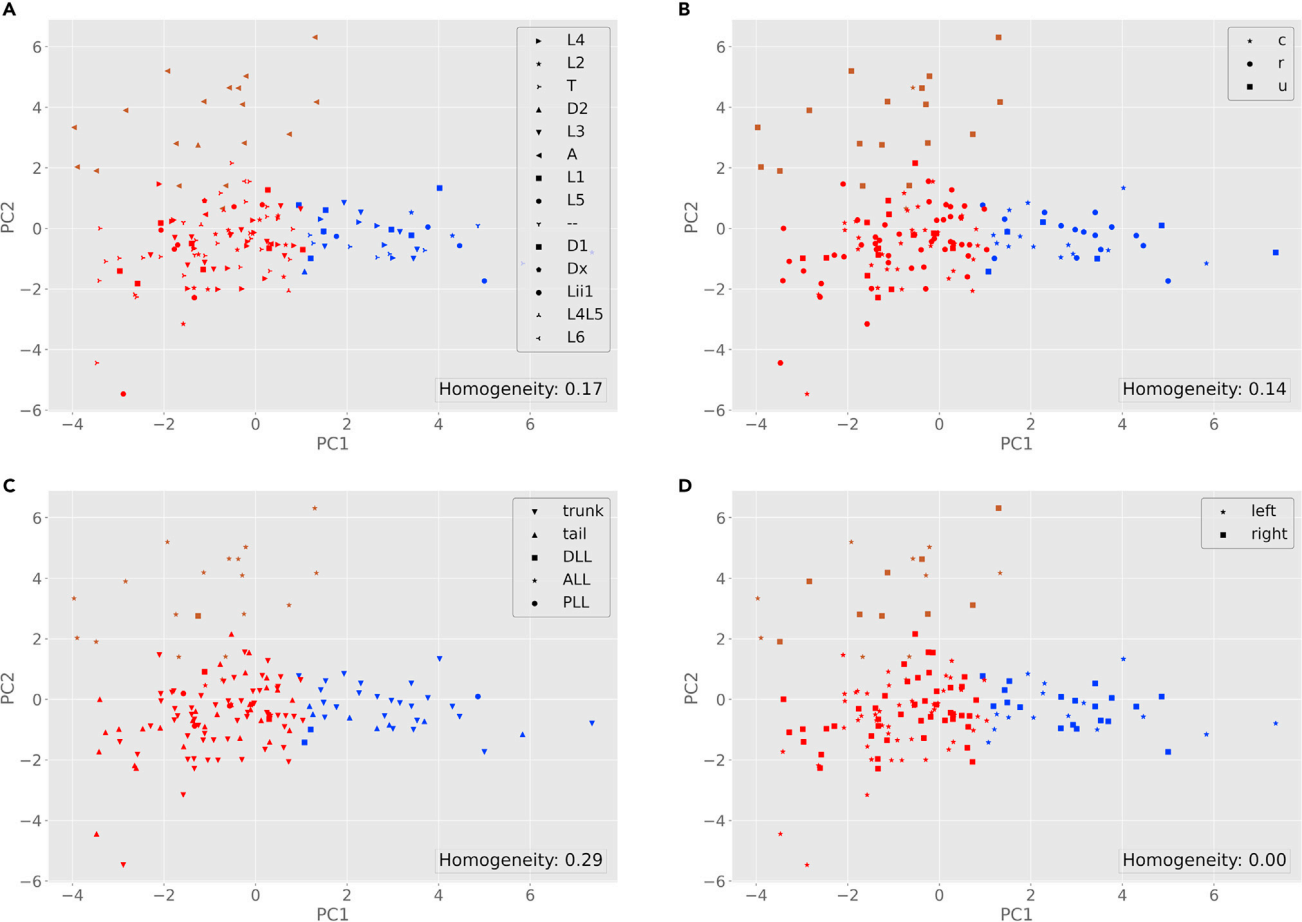
Visualization of the neurons based on K-means results (color-coded clusters) and their ground truth labels (shapes) For label meanings, see Figure 5 legend.
  c. Homogeneity is a clustering metric based on the ground truth labels. It checks if clusters contain only samples belonging to a single class. A clustering result satisfies homogeneity if all of its clusters contain only data points that are members of their ground truth class. This metric is bounded between 0 and 1, with lower values indicating low homogeneity (Rosenberg and Hirschberg, 2007).
  d. You can alter the input parameters before running the python script.
15. From your Python environment or command line, run ‘python labeled-GMM.py -i ./Data/neuron_features.csv -l ./Data/neuron_labels.csv -k 3’.
  a. This script reads the neuronal data and groups them into 3 clusters, just as in steps 9a-d above.
  b. The program then outputs the homogeneity score (as described in 14.c) and a series of scatter plots, each displaying the color-coded distributions with symbols (shapes) corresponding to the true class labels in each label category (Figure 7).

**Figure 7:**
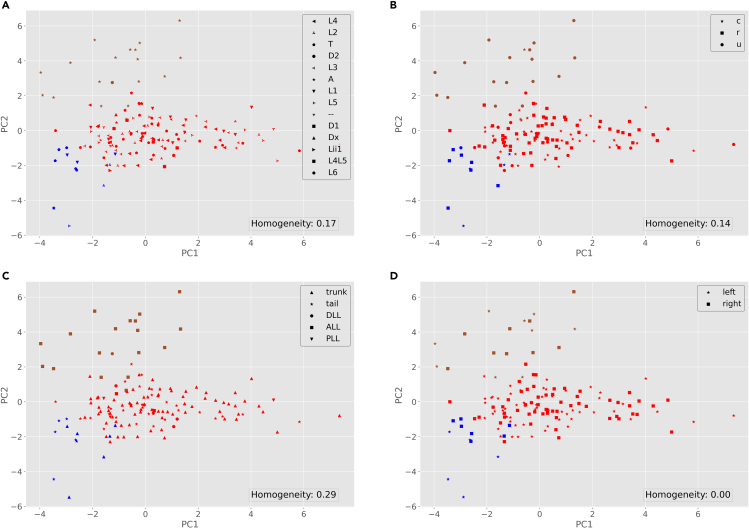
Visualization of the neurons based on GMM results (color-coded groups) and their ground truth labels (shapes) For label meanings, see Figure 5 legend.
  c. You can alter the input parameters before running the python script.

**Figure 8:**
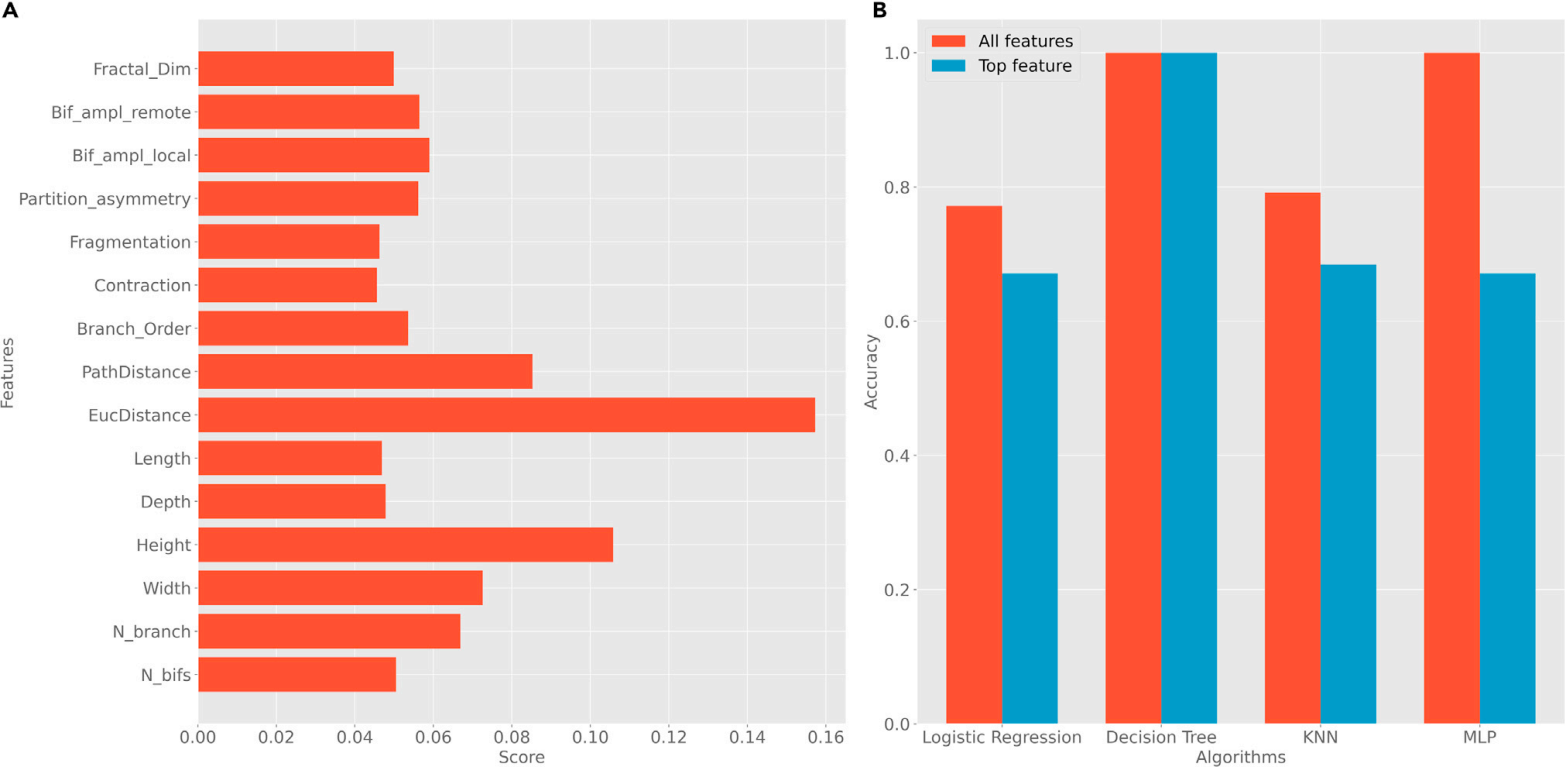
Supervised analysis results (A) Feature importance of the data. (B) Classification accuracy of logistic regression, decision tree, K-nearest-neighbor (K-NN), and multilayer perceptron (MLP) using all features and just the top feature. For more information on the morphological features see step 7 and for details on feature importance see step 16.

**Figure 9:**
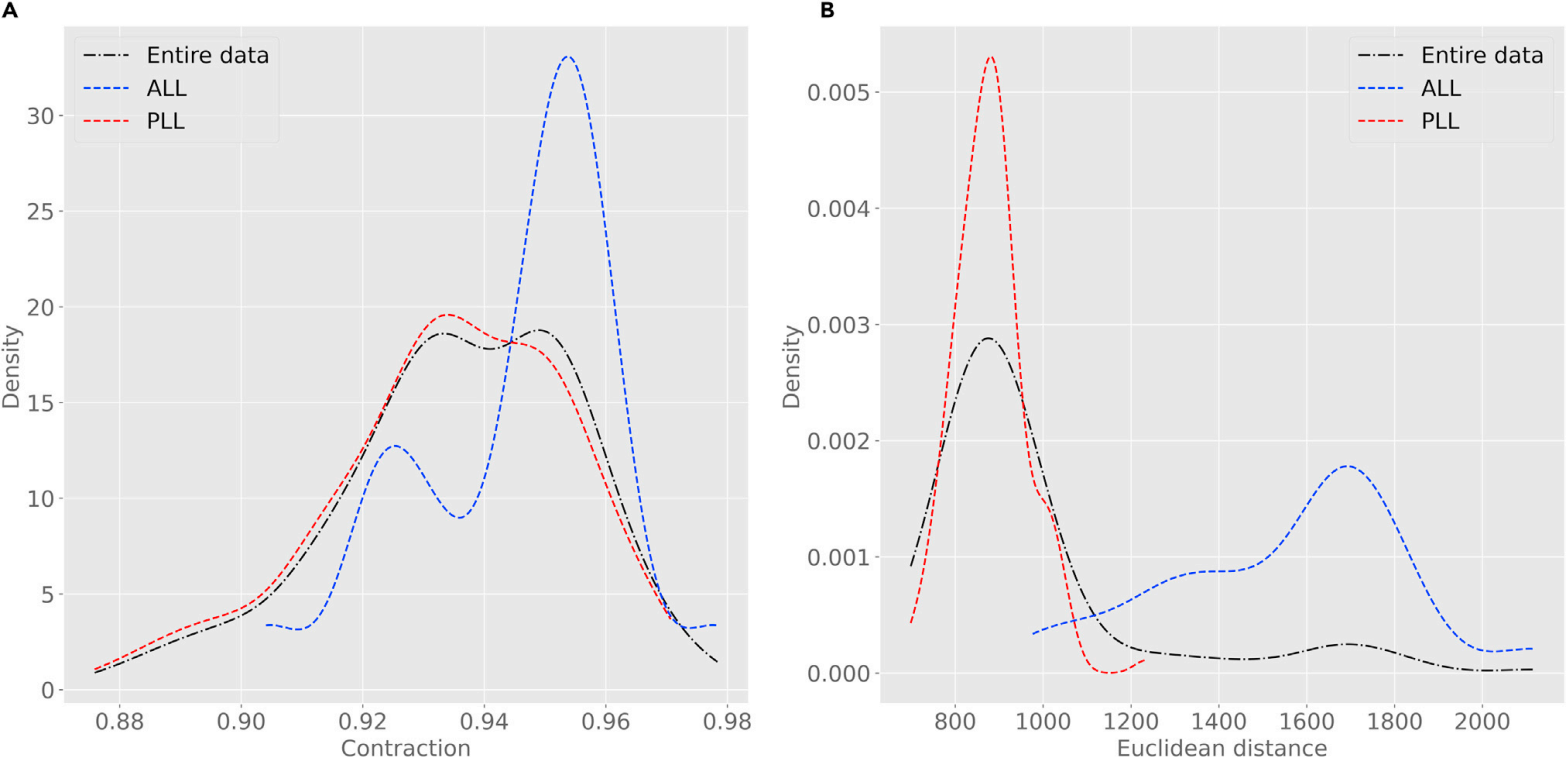
Density analysis Density plot for features ‘Contraction’ (branch tortuosity) (A) and ‘EucDistance’ (maximum straight distance from soma to tips) (B) in the entire dataset as well as in selected sub-class labels (ALL and PLL).

**Figure 10:**
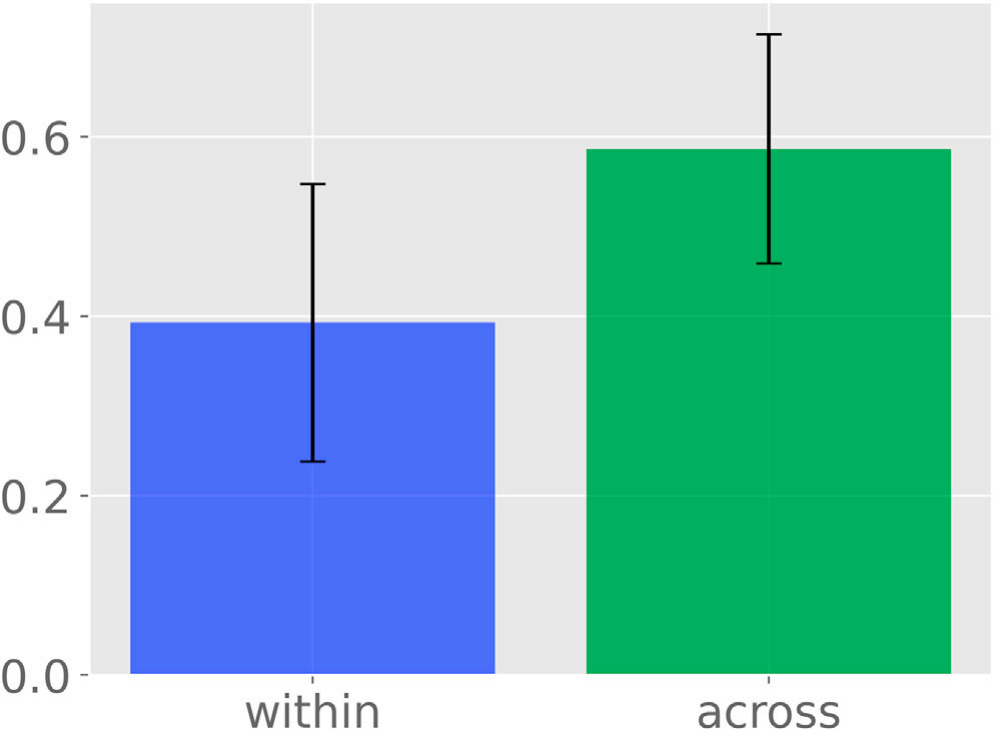
Statistical analysis of persistence diagram vectors (PDVs) relative to lateral lines (LL) The script first calculates the pairwise arccosine distances between PDVs of ‘within’ and ‘across’ populations with respect to class labels (ALL and PLL), and then performs their statistical comparison. Bar plot shows the average of ‘within’ and ‘across’ distances with error bars indicating standard deviations.

***Note:*** Model-specific parameters (e.g., number of clusters) will be inserted by the user through the command prompt and users will be guided with appropriate help messages from the command prompt in the clustering and classification algorithms. Furthermore, users should be advised that we have accepted the default python package parameters (e.g., number of iterations, penalties, and learning rates) offered by the Scikit-learn toolkit. Please refer to the Scikit-learn package manuals to check if the default settings are suitable for your data.

16. From your Python environment or command line, run ‘**python supervised-classification.py -i ./Data/neuron_features.csv -l ./Data/neuron_labels.csv’** and follow the command line instructions.
  a. This script reads and pre-processes the neuronal data and their corresponding labels from the data folder.
  b. The script asks which class label you want to select for the task of classification. This could vary based on your choice of dataset.
  c. The program then calculates the feature importance using the ‘ExtraTreesClassifier’ algorithm (see Note below) and plots those values in a bar graph.
    ***Note:*** The ExtraTreesClassifier algorithm used in step 16 is an approach to feature selection based on the Random Forest method. Random Forest creates several tree-based models using the features and attempts to classify the data via those decision trees. The importance of the features depends on the number of times that feature is used to split a node and on the number of samples it splits. Specifically, this algorithm weighs the total decrease in “node impurity” by the proportion of samples reaching that node. A Mean Decrease in Impurity (MDI) is then calculated as the average of this measure over all trees of the ensemble. In other words, the fewer splits a feature needs to classify the data, and the larger the proportion it classifies, the “purer”, hence more important, it is (Geurts et al., 2006).
  d. Next, the program trains four distinct machine learning algorithms on the labeled data: ‘logistic regression’, ‘decision tree’, ‘k-nearest-neighbors’, and ‘multilayer perceptron’.
  e. The program then calculates and plots the accuracy of each of those four algorithms in two different scenarios: if considering all morphological attributes of the neurons; and if only considering the most important attribute.
  f. Here we show a representative classification example for the class label ‘region’ (Figure 8). To obtain the results for other dimensions, follow command line instructions and change ‘class label’ when the command prompt asks for the class label.

17. From your Python environment or command line, run ‘**python supervised-density.py -i ./Data/neuron_features.csv -l ./Data/neuron_labels.csv’** and follow the command line instructions.fakn
  a. This script reads and pre-processes the neuronal data and their corresponding labels from the data folder.
  b. The script then asks which feature and which class label you want to select for the density analysis. This varies based on your choice of dataset.
  c. In this example, we have selected the morphological features ‘Contraction’ and ‘EucDistance’ to run density analysis. Nevertheless, the analysis could be repeated on any other dimension of the data. To do so, follow the command line instructions.
  d. Afterward, from the command line instructions we select ‘lateral line (LL)’ class labels to perform density analysis within this class for different class labels, namely, ‘ALL’ and ‘PLL’ for anterior and posterior LL, respectively..
  e. The program plots the density curve for the ‘Contraction’ and ‘EucDistance’ data considering different ‘lateral line’ class labels (Figure 9).

18. From your Python environment or command line, run ‘**python supervised-pdv-comparison.py -p ./Data/PDVs -l ./Data/neuron_labels.csv’** and follow the command line instructions.
  a. This script reads the persistent diagram vectors and the data labels from the data folder (see Note below).
    ***Note:*** The persistence diagram vectors (PDVs) utilized in step 18 are based on the concept of topological persistence in the field of computational topology (Kanari et al., 2018). PDVs are derived from a simplified representation of the neural arbor that only considers the stems, bifurcations, and terminations while ignoring all continuation points along the branches. In other words, this process creates a straight segment between any two topological nodes of the tree representing the Euclidean length of each branch. The descriptor function is the total length of the unique path (i.e., the sum of all branch lengths) from the tree stem to any point in the tree. The stem is the tree root, typically corresponding to its point of emergence from the soma (Li et al., 2017). Starting from this simplified representation, persistence analysis sweeps the neuron tree in decreasing function values, i.e., beginning from the farthest terminal tip, while tracking the appearance (end point) and disappearance (start point) of each neurite branch. The persistence diagram summarizes all resulting appearances and disappearances into a set of 2D points whose (x, y) coordinates represent the distance from the soma of the end and start points of each branch. Mathematically, the set of points in the persistence diagram captures a nested branch decomposition of the neuron tree with respect to the simplified Euclidean branch length description. Finally, the persistence diagram summary is converted into a 100-dimensional vector representing the function values at 100 positions sampled uniformly between the minimum and maximum values, corresponding respectively to the beginning of the tree and the farthest terminal tip. Intuitively, persistence diagram vectors capture the morphological information similar to that represented in Sholl diagrams (Garcia-Segura and Perez-Marquez, 2014; Sholl, 1953). The advantage of this approach, however, is that it produces a proper metric space which allows quantitative applications such as those illustrated in this protocol. PDVs for the data used in this analysis are available on NeuroMorpho.Org. For instructions to generate PDVs from SWC files or additional information on their interpretation, refer respectively to the corresponding GitHub page https://github.com/Nevermore520/NeuronTools or to the Frequently Asked Question entry of NeuroMorpho.Orghttp://neuromorpho.org/myfaq.jsp?id=qr11
  b. The script then asks which feature, class label, and values you want to select for the density analysis. This varies based on your choice of dataset.
  c. In this example, we illustrated the statistical analysis on lateral line (LL) to compare class labels ‘ALL’ and ‘PLL’. Nevertheless, the comparison could be repeated for any other class labels. To do so, follow the command line instructions and provide your desired input values.
  d. The program calculates pairwise arccosine distances of different vectors based on their labels and groups them in ‘within’ or ‘across’ populations for same labels (ALL/ALL or PLL/PLL) and different labels (ALL/PLL), respectively.
  e. Afterward, the software runs student’s t-test and displays a statement indicating whether the two groups (‘within’ vs ‘across’) are statistically different based on the resulting p-value.
  f. The program also outputs a bar plot of the average value of ‘within’ and ‘across’ populations with error bars indicating S.D. (Figure 10).

***Optional:*** To disregard some of the data points from the visualization or analysis based on their class or relevance, simply remove the corresponding entries from both the ‘neuron_features.csv’ and ‘neuron_labels.csv’ files. Save a copy of the original files prior to any modifications.

## Expected outcomes

In this protocol, neuronal reconstructions are quantified into measurable attributes expressed as numerical values. Moreover, the digitally reconstructed neurons are compared through unsupervised and supervised analysis, manifesting the most distinguishing features conducive to neuronal classification.

## Limitations

The protocol only includes morphological attributes of the neural reconstructions into account. Future variations could consider additional features in the analysis, such as genetic expression or time-lapse dynamics (Nanda et al., 2020).

## Troubleshooting

Use specified version of the tools or packages for proper functioning. Re-install tools if errors persist.

### Problem 1

Different Python packages overlap causing code to malfunction.

### Potential solution

Install Python in a virtual environment and install a fresh copy of the mentioned packages. For further details on how to install a virtual environment for different operating systems please visit https://packaging.python.org/guides/installing-using-pip-and-virtual-environments/ (Step 2 in before you begin)

### Problem 2

L-Measure does not produce the desired outputs (Step 1).

### Potential solution

Make sure the software is executable and has appropriate privileges. In Linux, for example, this is achieved using the “chmod u+x Lm.∗” command.

### Problem 3

Depending on your data, during the classification phase you might receive a warning that some of the algorithms did not converge yet. For example, after running the supervised_classification.py script you might see a warning like: *(ConvergenceWarning: Stochastic Optimizer: Maximum iterations (90) reached and the optimization hasn't converged yet. warnings.warn) (Step 16)*

### Potential solution

In such cases, you need to increase the number of iterations for that algorithm. For example, in the case above, in the multi-layer-perceptron algorithm, increase the ‘max_iter’ parameter on line 143 of the supervised_classification.py script with a text editor of your choice.

### Problem 4

Issue running python scripts while providing input parameters (any step).

### Potential solution

All python scripts are enhanced with help command inside. Run ‘python [Script].py -h’ to see more information about the input parameters of the script.

### Problem 5

Python runs into error while reading files from the provided paths (any step)

### Potential solution

Check path names and file names to make sure they exist in the appropriate location and their name does not include any special characters.

## Resource availability

### Lead contact

Further information and requests for resources should be directed to and will be fulfilled by the lead contact, Giorgio A. Ascoli (ascoli@gmu.edu).

### Materials availability

This study did not generate any reagents.

## Data Availability

All image stacks, data, and scripts of this protocol are publicly available on GitLab at https://gitlab.orc.gmu.edu/kbijari/zebrafish-analysis-protocol/tree/master/. Neuronal reconstructions are available at NeuroMorpho.Org (Lopez-Schier archive)
